# *GhHAI2*, *GhAHG3,* and *GhABI2* Negatively Regulate Osmotic Stress Tolerance *via* ABA-Dependent Pathway in Cotton (*Gossypium hirsutum* L.)

**DOI:** 10.3389/fpls.2022.905181

**Published:** 2022-05-19

**Authors:** Hamna Shazadee, Nadeem Khan, Lu Wang, Xinyu Wang

**Affiliations:** ^1^College of Life Sciences, Nanjing Agricultural University, Nanjing, China; ^2^Agriculture and Agri-Food Canada, Ottawa Research and Development Centre, Ottawa, ON, Canada; ^3^Department of Biology, University of Ottawa, Ottawa, ON, Canada

**Keywords:** PP2Cs, osmotic tolerance, cotton, VIGS, abscisic acid

## Abstract

The type 2C protein phosphatases (PP2Cs) are well known for their vital roles in plant drought stress responses, but their molecular mechanisms in cotton (*Gossypium hirsutum* L.) remain largely unknown. Here, we investigated the role of three clade A *PP2C* genes, namely, *GhHAI2*, *GhAHG3,* and *GhABI2*, in regulating the osmotic stress tolerance in cotton. The transcript levels of *GhHAI2*, *GhAHG3,* and *GhABI2* were rapidly induced by exogenous abscisic acid (ABA) and polyethylene glycol (PEG) treatment. Silencing of *GhHAI2*, *GhAHG3,* and *GhABI2 via* virus-induced gene silencing (VIGS) improved osmotic tolerance in cotton due to decreased water loss, increase in both relative water content (RWC) and photosynthetic gas exchange, higher antioxidant enzyme activity, and lower malondialdehyde (MDA) content. The root analysis further showed that *GhHAI2*, *GhAHG3,* and *GhABI2*-silenced plants were more responsive to osmotic stress. Yeast two-hybrid (Y2H) and luciferase complementation imaging (LCI) assays further substantiated that GhHAI2, GhAHG3, and GhABI2 interact with the core receptors of ABA signaling, GhPYLs. The expression of several ABA-dependent stress-responsive genes was significantly upregulated in *GhHAI2-*, *GhAHG3-,* and *GhABI2*-silenced plants. Our findings suggest that *GhHAI2*, *GhAHG3*, and *GhABI2* act as negative regulators in the osmotic stress response in cotton through ABA-mediated signaling.

## Introduction

Plants are constantly exposed to diverse environmental conditions, such as water deficit, high salinity, flooding, and extreme temperatures, which adversely affect their growth and productivity (Claeys and Inzé, 2013). These abiotic stresses greatly impact plant morphology and cause damage to plant cells. For instance, excessive accumulation of reactive oxygen species (ROS) affects cell membrane stability, reduces photosynthetic efficiency, accelerates protein deformation, and causes leaf wilting (Hanin et al., 2011; Choudhury et al., 2013, 2017). As plants are sessile in nature, various complex mechanisms have been developed to respond appropriately to such harmful conditions. One of these mechanisms is the induction of diverse number of stress-responsive genes (Nakashima and Yamaguchi-Shinozaki, 2006). Previous studies have reported many stress-induced proteins, such as enzymes involved in the ABA signaling pathway, protein phosphatases, numerous protein kinases, osmotic adaptive proteins, cellular protective enzymes, and transcription factors (Zhu, 2002, 2016).

The type 2C protein phosphatases (PP2Cs) belong to a major group of gene families known to mediate abiotic stress signaling pathways and signal transduction processes. PP2Cs have been identified as the first key component of the ABA signaling pathway (Koornneef et al., 1984). The phytohormone ABA plays a vital role in regulation of plant drought stress responses (Yoshida et al., 2014; Zhu, 2016). Genetic studies have reported a canonical mechanism underlying ABA signal transduction in *Arabidopsis thaliana*. Under water deficit conditions, plants exhibit an elevated level of ABA which is perceived by the pyrabactin resistance 1 (PYR1)/PYR1-like (PYL)/regulatory component of the ABA receptor (RCAR) protein family (Ma et al., 2009; Park et al., 2009). In the absence of ABA, clade A PP2Cs interact with sucrose nonfermenting 1-related protein kinase 2 (SnRK2), thereby preventing their activity by dephosphorylation. ABA perception leads to the binding of PYL receptors to PP2Cs, which release SnRK2 for subsequent phosphorylation of ABA-Responsive Element (ABRE) Binding Factors (ABFs) which regulate transcription of ABA-responsive genes (Cutler et al., 2010; Nakashima and Yamaguchi-Shinozaki, 2013).

In recent years, several studies have functionally characterized PP2C family members, for example, in *Arabidopsis*, nine PP2C group A members have been identified as component of ABA signaling pathway; *ABI1* (ABA insensitive1), *ABI2*, *HAB1* (hypersensitive to ABA1), *HAB2*, *AHG1* (ABA-hypersensitive germination1), *AHG3/AtPP2CA*, *HAI1* (highly ABA-induced) *HAI2,* and *HAI3* (Saez et al., 2004; Schweighofer et al., 2004; Rubio et al., 2009; Antoni et al., 2012; Kim et al., 2013; Rodrigues et al., 2013; Née et al., 2017; Yoshida et al., 2019). Double mutant plants of *abi1* and *abi2* were responsive to ABA, suggesting that *ABI1* and *ABI2* function negatively in ABA signaling pathway (Merlot et al., 2001). Under ABA treatment, *ahg1* and *ahg3* double mutants displayed stronger phenotypes than single parental mutant, implying that *AHG1* and *AHG3* function together in regulating ABA signaling pathway (Nishimura et al., 2007). Similarly, all members of *PP2C-A* were upregulated when subjected to exogenous ABA; however, *HAI1*, *HAI2,* and *HAI3* expressions were strongly induced in vegetative phase (Fujita et al., 2009). Likewise, transgenic *Arabidopsis* overexpressing rice clade A *OsPP108* showed enhanced tolerance under salt, mannitol, and drought stress, but reduction in ABA sensitivity (Singh et al., 2015). The overexpression of *OsABIL2,* which encodes another rice clade A of *PP2C*, exhibits ABA insensitivity and significantly altered phenotypes, such as stomatal density and root architecture, leading to the drought hypersensitivity (Li et al., 2015). Similarly, maize *ZmPP2C-As* were recently characterized and their role in drought tolerance were elucidated. For instance, *Arabidopsis* plants overexpressing *ZmPP2C-2A* and *ZmPP2C-6A* were sensitive to drought stress, suggesting their negative role in drought stress response (He et al., 2019). Also, transgenic studies in maize and *Arabidopsis* verified that *ZmPP2C-A10* function as negative regulator of drought tolerance (Xiang et al., 2017). So far, using bioinformatics analysis, the *PP2C* gene family has been identified in several species, including *Arabidopsis* (Schweighofer et al., 2004), rice (Xue et al., 2008), cotton (Shazadee et al., 2019), wheat (Yu et al., 2019), soybean (Chen et al., 2018), and Chinese cabbage (Khan et al., 2019). However, the functional characterization of *PP2C* genes in cotton remains largely obscure.

Cotton (*Gossypium hirsutum* L.) is one of the most important fiber and oil crops, commercially grown worldwide. Various abiotic stresses particularly drought stress greatly affect cotton growth and limit fiber yield and lint quality, resulting in a significant production losses (Pettigrew, 2004). Moreover, 57% of global cotton is grown in regions with high water stress (WRI, 2013). Thus, development of drought resistant cotton cultivars and improving water use efficiency are crucial to sustain the cotton industry. In a previous study, we identified 18 Clade A PP2Cs in cotton (*G. hirsutum*; Shazadee et al., 2019) but their functional role was unexplored. It has been recently reported (Lu et al., 2019) and observed that the expression of clade A *PP2Cs* is highly induced under osmotic and ABA treatment in cotton. These observations suggest a role of cotton clade A PP2Cs in abiotic stress; however, no stress-related phenotype has been associated with group A PP2Cs in cotton yet. Hence, it is important to further investigate the molecular mechanism of clade A PP2Cs in response to drought stress in cotton.

In this study, we characterized three members of clade A *PP2Cs*; *GhHAI2, GhAHG3,* and *GhABI2* in cotton in order to investigate their roles in drought tolerance. The *GhHAI2, GhAHG3,* and *GhABI2* were highly induced by ABA and PEG treatment. Silencing of *GhHAI2*, *GhAHG3,* and *GhABI2* improved osmotic tolerance of cotton plants. Yeast two-hybrid (Y2H) and luciferase complementation imaging (LCI) assays revealed that GhHAI2, GhAHG3, and GhABI2 interact with GhPYLs and regulate ABA signaling pathway. Furthermore, we demonstrated that *GhHAI2-*, *GhAHG3-,* and *GhABI2*-silenced plants increased the expression of ABA-dependent stress-responsive genes. In brief, our results suggest that *GhHAI2*, *GhAHG3,* and *GhABI2* function as crucial negative regulators in osmotic stress response by an ABA-dependent pathway indicating their potential roles in drought tolerance.

## Materials and Methods

### Plant Material and Stress Treatment

For expression analysis of *GhHAI2*, *GhAHG3,* and *GhABI2* in cotton, seedlings were planted in Hoagland nutrient solution with a 16 h/8 h light/dark cycle at 25°C. Then, three-week-old seedlings were subjected to 15% PEG 6000 for osmotic treatment. For ABA treatment, the plants were sprayed with 200 μm ABA. Leaves were collected after stress treatment at the designated time (0, 6, 12, 24, and 48 h).

For evaluation of osmotic stress tolerance, *G. hirsutum*, cultivar Xinluzao, plants were used to perform the experiments. Cotton plants were grown in a growth chamber under a 16 h/8 h light/dark cycle at 25°C. Pot-grown cotton seedlings at two true leaves stage were treated with 15% PEG for osmotic stress, and in parallel, water treatment was used as mock control. After PEG stress, plant leaves were collected at different time points, immediately frozen in liquid nitrogen and stored at −80°C until further use. Root assay was performed after 7 days of osmotic stress. Each of the experiments was performed in triplicate.

### Cloning and Sequence Analysis of GhHAI2, GhAHG3, and GhABI2

We obtained the full-length ORFs of *GhHAI2*, *GhAHG3,* and *GhABI2 via* PCR; the primers were designed using the coding sequence of *GhHAI2*, *GhAHG3,* and *GhABI2* (Supplementary Table S1). Alignment of cotton GhHAI2, GhAHG3, and GhABI2 and *Arabidopsis* HAI2, AHG3, and ABI2 was performed with ClustalW (Thompson et al., 1994). The MEGA program (version 7.0) was used to construct the phylogenetic tree *via* the Neighbor-Joining (NJ) method and 1,000 bootstrap replications.

### Subcellular Localization

The coding regions of *GhHAI2*, *GhAHG3,* and *GhABI2* were amplified by PCR and inserted into the pBin-GFP4 (green fluorescent protein) expression vector. The three vectors were separately introduced into *Agrobacterium tumefaciens* strain GV3101 cells and transiently expressed in *Nicotiana benthamiana* leaf cells *via A. tumefaciens* infiltration method. After 3 days of infiltration, fluorescence signals were detected using a confocal laser-scanning microscope (Zeiss, LSM710).

### *Agrobacterium tumefaciens-*Mediated VIGS

Inserts to generate TRV2:*GhHAI2*, TRV2:*GhAHG3*, TRV2:*GhABI2,* and positive control TRV2:*GhCLA1* were amplified from *G. hirsutum* cultivar Xinluzao cDNA with primers containing the restriction sites *Eco*RI and *Xho*I. The primers for cloning are listed in Supplementary Table S1. Vectors constructed in binary tobacco rattle virus (TRV) vector, including pTRV1 and pTRV2 (*GhHAI2*, *GhAHG3,* and *GhABI2* and *GhCLA1*), were introduced into *A. tumefaciens* strain GV3101 by electroporation. The Agrobacteria culture carrying the above pTRV1 and pTRV2 constructs was infiltrated into two fully expanded cotyledons of seven-day-old cotton plants using a needle-less syringe as previously described (Gao et al., 2011, 2013). The *GhCLA1* construct was used as a visual marker to determine VIGS efficiency. After 14 days of Agrobacteria inoculation, the silenced plants were subjected to 15% PEG treatment for the indicated times. VIGS experiments were repeated three times with more than 30 plants for each construct per replicate.

### Measurement of Water Loss and RWC

For relative water content (RWC) measurement, six leaves were detached from individual cotton plants and the fresh weight (FW) was recorded. To record turgid weight (TW), the leaves were soaked in distilled water for 4 h at room temperature with constant light. The leaves were then dried at 65°C for 24 h to obtain the dry weight (DW). RWC was calculated using the formula: RWC (%) = [(FW−DW)/(TW−DW)] × 100. To measure water loss, aerial parts of six cotton seedlings were detached and placed on clean filter paper on a laboratory bench. At various time intervals, the total FW was recorded. Water loss was calculated as the decrease in fresh weight as a percentage of the initial fresh weight of the detached seedlings parts. Both assays were performed in three biological repeats.

### Gas Exchange

Gas exchange measurements were taken from three-week-old cotton plants under normal and PEG conditions. The photosynthetic rate (*A*, μmol CO_2_ m^−2^ s^−1^), stomatal conductance (*g*s, mol H_2_O m^−2^ s^−1^), intercellular CO_2_ concentration (*Ci*, μmol CO_2_ mol^−1^), and transpiration rate (*E*, mmol H_2_O m^−2^ s^−1^) were measured with a portable photosynthesis system Li-6400XT (Li-COR Inc., United States) under 1,500 μmol m^−2^ s^−2^ light intensity, 23°C ± 2°C temperature, and 300 μmol mol^−1^ CO_2_ concentration. For each gene, at least seven biological replicates per treatment were measured.

### Measurement of Antioxidant Enzymes and MDA Content

Fresh leaves of cotton plants under normal and PEG conditions were used for the measurement of antioxidant enzymes activity and Malondialdehyde (MDA) content. The peroxidase (POD; U mg^−1^ protein), superoxide dismutase (SOD; U mg^−1^ protein), catalase (CAT; U mg^−1^ protein) activities, and MDA (nmol g^−1^) content were determined using analytical kits (Nanjing Jiancheng Bioengineering Institute, Nanjing, China) following the manufacturer’s protocol as described (Kumar et al., 2020). Three biological replicates were used to investigate each physiological index.

### RNA Extraction, First-Strand cDNA Synthesis, and qPCR

Total RNA was extracted from various organs of cotton plants using Biospin plant Total RNA Extraction Kit (Bioer technology, Hangzhou, China) according to the manufacturer’s protocol. gDNase-treated RNA was reverse transcribed to generate first-strand cDNA using Prime Script^™^ RT Reagent Kit (TaKaRa, United States). Gene expression levels were determined using qPCR assay, which was conducted with SYBR^®^ Premix Ex Taq^™^ (TaKaRa, United States) and an ABI 7300 qPCR System (Applied Biosystems, CA, United States). The real-time PCR amplification reactions are briefly described in our previously reported study (Shazadee et al., 2019). The 2^-ΔΔCt^ method was used to determine relative expression level (Livak and Schmittgen, 2001). Cotton histone3 (*AF024716*) gene was used as an internal control. All the primers were designed using Primer Blast in NCBI (Supplementary Table S1). Three biological replicates, each containing three technical replicates, were used for each sample.

### Y2H Assay

The Y2H assay was based on the Matchmaker GAL4 two-hybrid system (Clontech, United States). The *GhHAI2, GhAHG3,* and *GhABI2* and *GhPYLs* coding regions were independently cloned into the pGBKT7 (BD) and pGADT7 (AD) vectors, respectively. The construct pairs were co-transformed into AH109 yeast strain cells and grown on SD/−Trp/−Leu/-His/−Ade (SD/-QDO) medium containing 5-bromo-4-chloro-3-indolyl-alpha- D-galactopyranoside (X-α-gal). Photographs of resulting yeast growth were taken 3 days after incubation.

### LCI Assay

For luciferase complementation imaging (LCI) assay, the open reading frames of *GhHAI2, GhAHG3,* and *GhABI2* and *GhPYLs* without stop codons were cloned into pCAMBIA1300-CLuc or pCAMBIA1300-NLuc vectors using *Kpn*I and *Bam*HI or *Bam*HI and *Sal*I restriction sites, respectively. The fusion constructs were then transformed to *A. tumefaciens* GV3101. The suspensions were prepared by mixing the three *Agrobacterium* strains carrying the CLuc, and NLuc fusion and the gene silencing inhibitor p19 at a 1:1:1 ratio. For transient expression, the bacterial mixture was infiltrated into different locations on the same *N. benthamiana* leaves using a needle-less syringe. To measure LUC activity, 1 mM luciferin was sprayed into the leaves and kept in the dark for 5 min to quench the fluorescence. A low-light cooled CCD imaging apparatus was used to capture the LUC image at 3 min intervals.

### Statistical Analyses

All data are represented herein as the means ± standard deviations. The significant levels were determined by using Student’s *t-*test: ^*^p < 0.05; ^**^p < 0.01.

## Results

### Induction of *GhHAI2*, *GhAHG3,* and *GhABI2* After Exposure to PEG and ABA

**Figure 1:**
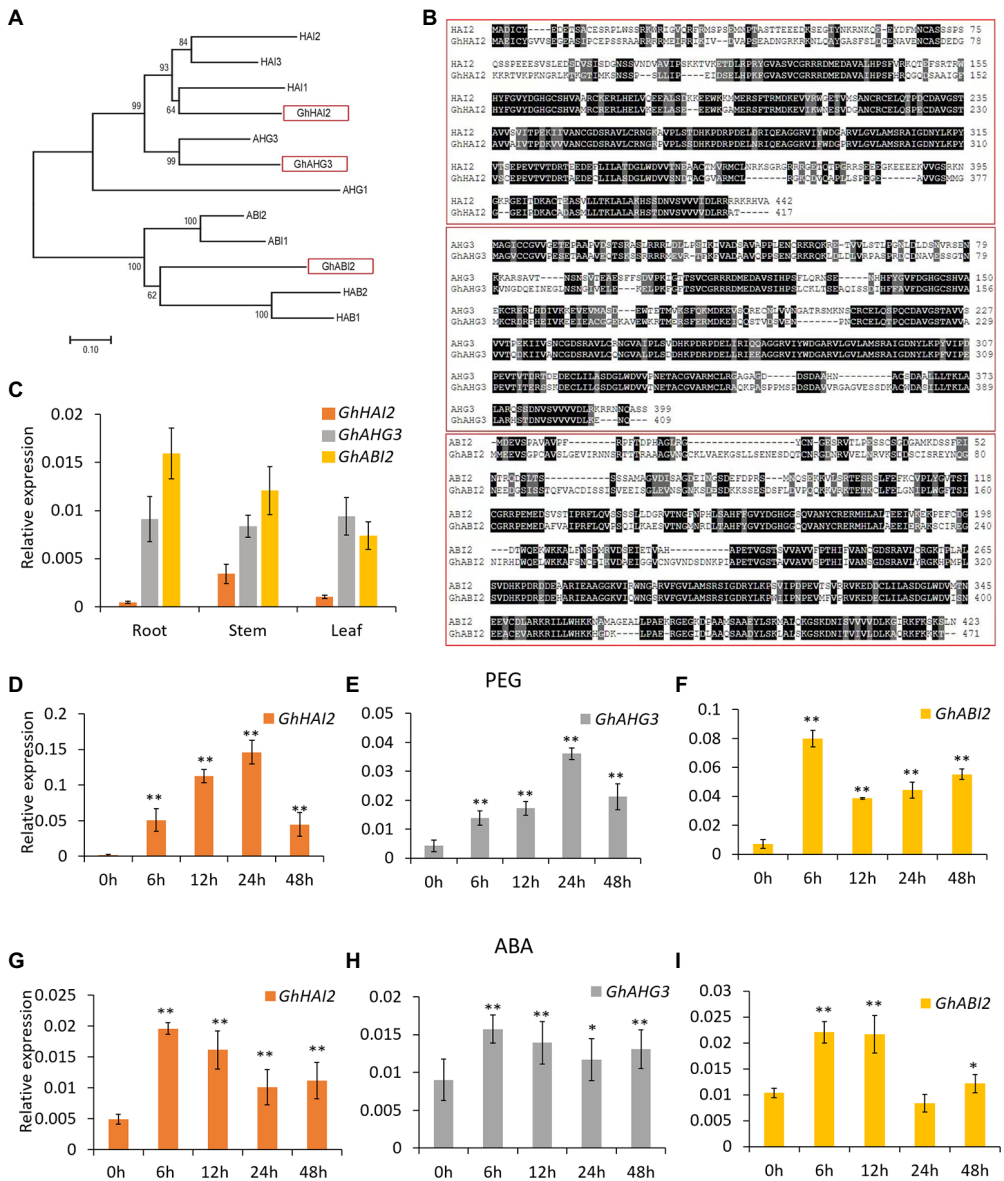
Sequence analysis and expression pattern of *GhHAI2*, *GhAHG3,* and *GhABI2* in different tissues and after exposure to PEG and ABA. **(A)** Phylogenetic analysis of GhHAI2, GhAHG3, and GhABI2 and *Arabidopsis* group A PP2Cs. The neighbor-joining tree was constructed using MEGA software (version 7.0) based on multiple alignments of GhHAI2, GhAHG3, and GhABI2 protein sequence and their homologous proteins in *Arabidopsis*. **(B)** Comparisons of deduced amino acid sequence of GhHAI2, GhAHG3, and GhABI2 with those of *Arabidopsis*. Identical amino acid residues are highlighted in black. **(C)** qPCR analysis of *GhHAI2*, *GhAHG3,* and *GhABI2* genes expression levels in three different tissues. **(D–F)** PEG- and **(G–I)** ABA-induced expression pattern of *GhHAI2*, *GhAHG3,* and *GhABI2* genes. Bars represent means ± standard deviation of three biological replicates. Asterisks represent statistically significant differences compared with untreated control (0 h; Student’s *t*-test; ^*^p < 0.05; ^**^p < 0.01).

In an RNA-seq assay and our previously reported study (Zhang et al., 2015; Shazadee et al., 2019), several *PP2C* genes were identified that were induced in response to osmotic stress. Of these candidate genes, we selected three strongly induced genes for their functional characterization in order to confirm their potential role in osmotic tolerance. Phylogenetic analysis revealed that these PP2Cs belong to clade A and share a close relationships with *Arabidopsis* HAI2, AHG3, and ABI2 (Figure 1A). Based on their similarity with *Arabidopsis*, we renamed them as GhHAI2, GhAHG3, and GhABI2, respectively. The deduced amino acid sequence of GhHAI2, GhAHG3, and GhABI2 encodes a protein of 417, 409, and 471 amino acids, and shares 56, 59, and 54% sequence similarity with *Arabidopsis* HAI2, AHG3, and ABI2, respectively (Figure 1B). The *Arabidopsis* clade A PP2Cs, HAI2, AHG3, and ABI2 have been known to function as negative regulators in ABA signaling pathway (Merlot et al., 2001; Leonhardt et al., 2004; Nishimura et al., 2007; Xue et al., 2008; Bhaskara et al., 2012). Therefore, we predicted that *GhHAI2*, *GhAHG3,* and *GhABI2* might show a similar expression pattern to *Arabidopsis HAI2*, *AHG3,* and *ABI2* and function in osmotic stress response in cotton.

qPCR analysis revealed that *GhHAI2*, *GhAHG3,* and *GhABI2* genes are constitutively expressed in root, stem, and leaf tissues; however, the expression level of *GhHAI2* was relatively lower (Figure 1C). To verify that *GhHAI2*, *GhAHG3,* and *GhABI2* respond to osmotic stress, we monitored their expression levels in cotton leaves after exposure to PEG and ABA using qPCR. Before treatment, the *GhHAI2*, *GhAHG3,* and *GhABI2* expressions were weakly induced. However, PEG and ABA triggered a significant accumulation of *GhHAI2*, *GhAHG3,* and *GhABI2* transcripts within 6–48 h (Figures 1D–I). These results suggested that *GhHAI2*, *GhAHG3,* and *GhABI2* expressions are strongly induced after PEG and ABA treatment indicating that these genes could function in response to dehydration in cotton.

### Subcellular Localization of GhHAI2, GhAHG3, and GhABI2

**Figure 2:**
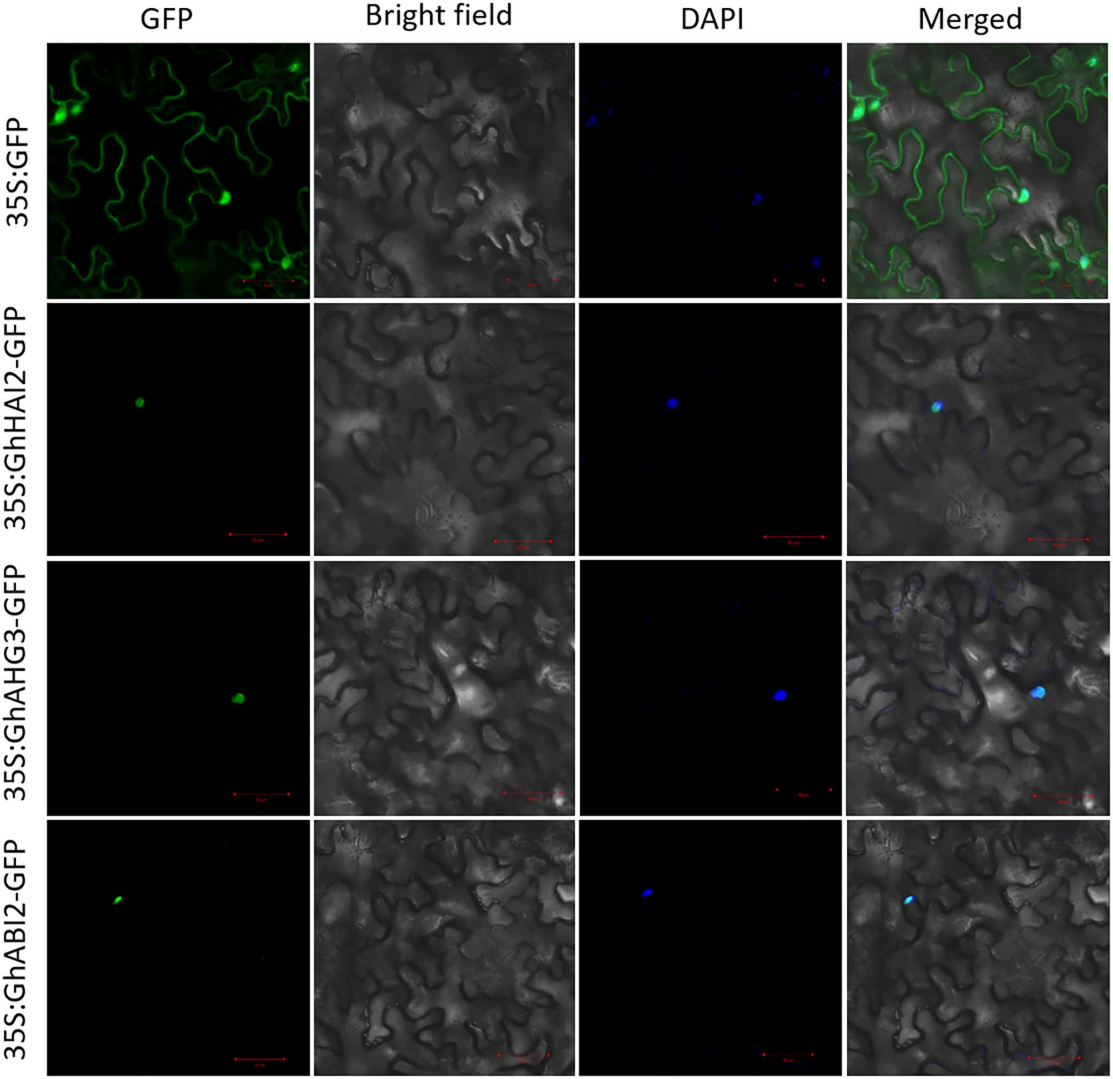
Subcellular localization of GhHAI2, GhAHG3, and GhABI2 based on the transient expression of green fluorescent (GFP) fusion protein in the epidermal cells of *N. benthamiana*. The 35S:GhHAI2*-*GFP, 35S:GhAHG3-GFP, and 35S:GhABI2-GFP constructs were expressed in the leaves of *N. benthamiana* using agroinfiltration method. The GFP signal was observed using a confocal laser-scanning microscope. The DAPI staining was used as nucleus marker.

To investigate the subcellular localization of GhHAI2, GhAHG3, and GhABI2, we fused their coding regions to the green fluorescent protein (GFP) reporter gene under the control of the 35S promoter. We found that the transient expression of the 35S:GhHAI2-GFP, 35S:GhAHG3-GFP, and 35S:GhABI2-GFP fusion proteins in *N. benthamiana* leaves generated GFP signals in the nucleus. Using the 4′,6-diamidino-2-phenylindole (DAPI) staining, we observed that the blue signal localized to the nucleus overlapped with the GFP signals (Figure 2). These results indicate that GhHAI2, GhAHG3, and GhABI2 have a functional role in the nucleus.

### Silencing of *GhHAI2*, *GhAHG3,* and *GhABI2* Confers Osmotic Stress Tolerance in Cotton

**Figure 3:**
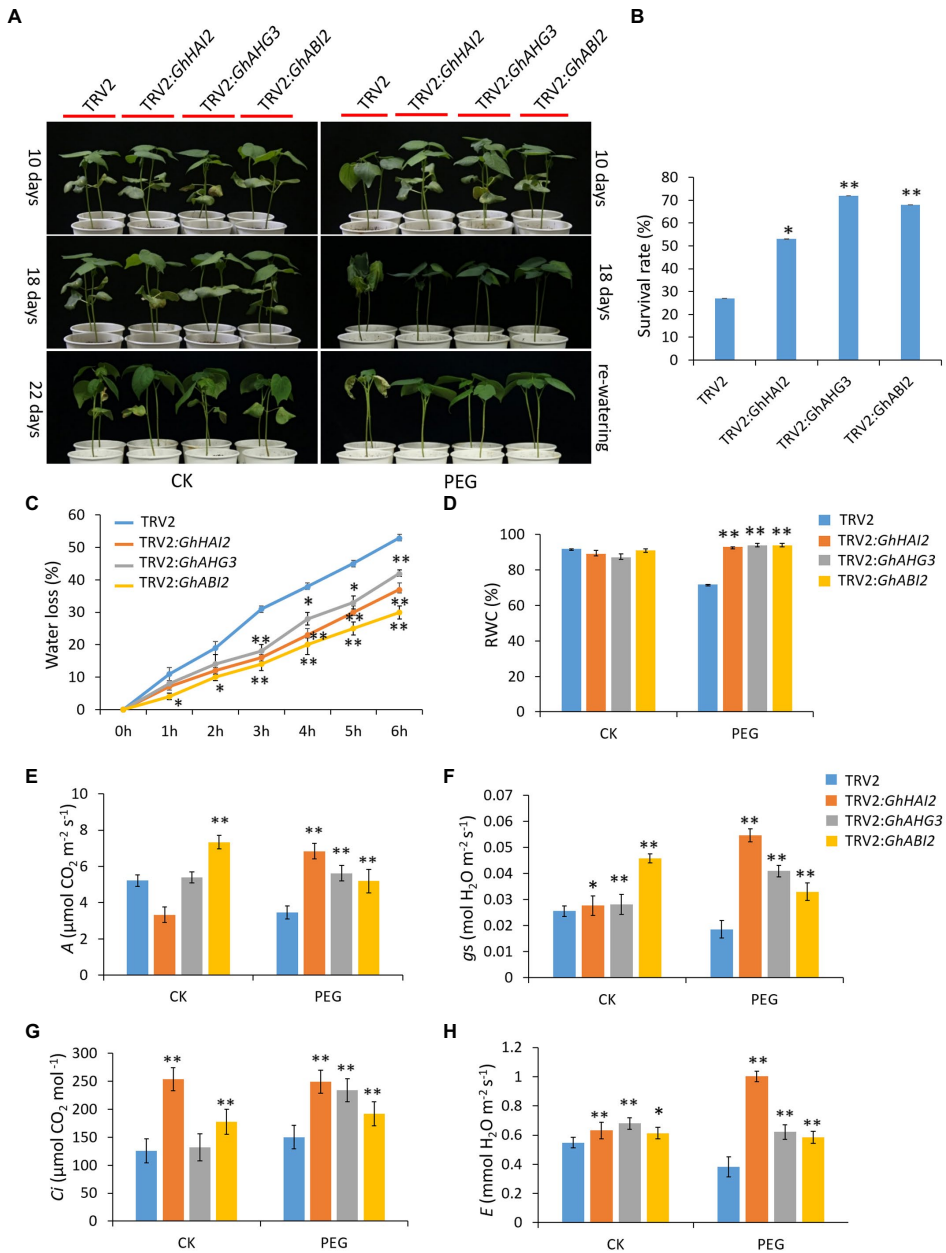
*GhHAI2*, *GhAHG3,* and *GhABI2* genes negatively regulate cotton osmotic response. **(A)** Phenotypic observations on the *GhHAI2-*, *GhAHG3-,* and *GhABI2*-silenced and control plants under normal (left panel) and PEG conditions (right panel). Three-week old plants were subjected to PEG treatment for 18 days and re-watered for 2 days. **(B)** Percentage of surviving plants after re-watering for 2 days. **(C)** Water loss from TRV2:*GhHAI2*, TRV2:*GhAHG3,* and TRV2:*GhABI2* and TRV2:00 seedlings under PEG conditions. **(D)** Relative water content (RWC) in the leaves of TRV2:*GhHAI2*, TRV2:*GhAHG3,* and TRV2:*GhABI2* and TRV2:00 plants under normal and PEG conditions. **(E–H)** Analyses of gas exchange parameters including, photosynthetic rate (*A*), stomatal conductance (*g*s), intracellular CO_2_ concentration (*Ci*), and transpiration rate (*E*) in TRV2:*GhHAI2*, TRV2:*GhAHG3,* and TRV2:*GhABI2* and TRV2:00 plants under normal and PEG conditions. Bars represent means ± standard deviation of three biological replicates. At least 30 plants were used for each biological replicate. Asterisks represent statistically significant difference between TRV2:*GhHAI2*, TRV2:*GhAHG3,* and TRV2:*GhABI2* and TRV2:00 plants (Student’s *t*-test; ^*^p < 0.05; ^**^p < 0.01).

To investigate the biological function of *GhHAI2, GhAHG3,* and *GhABI2* in responding to osmotic stress, we performed TRV-based virus-induced gene silencing (VIGS). Seven-day-old plants were hand-infiltrated using *Agrobacterium* cultures carrying VIGS vectors into cotton cotyledons. At 14 days post-infiltration (dpi), plants inoculated with TRV2:*GhCLA1*, a chlorophyll biosynthesis gene, exhibited obvious albino phenotype, which was uniformly distributed on entire true leaves (Supplementary Figure S1A). The expression levels of *GhHAI2*, *GhAHG3,* and *GhABI2* were significantly reduced in the silenced plants (TRV2:*GhHAI2*, TRV2:*GhAHG3,* and TRV2:*GhABI2*) than in control plants (TRV2:00; Supplementary Figures S1B–D). Subsequently, the VIGS plants were subjected to 15% PEG for 18 days and then re-watering for two days. Under well-watered conditions, no obvious difference was observed between TRV2:00 and TRV2:*GhHAI2*, TRV2:*GhAHG3,* and TRV2:*GhABI2* plants. However, after 10 days of PEG stress, TRV2:00 plants displayed serious wilting sooner than TRV2:*GhHAI2*, TRV2:*GhAHG3,* and TRV2:*GhABI2* plants. After further 8 days of water deficit condition and re-watering, *GhHAI2-*, *GhAHG3-,* and *GhABI2*-silenced plants displayed a stronger osmotic-tolerant phenotype in comparison with control plants (Figure 3A). At two days after re-watering, the survival rates of TRV2:*GhHAI2*, TRV2:*GhAHG3,* and TRV2:*GhABI2* plants were 53, 72, and 68%, respectively, whereas only 27% of TRV2:00 plants survived (Figure 3B). To determine whether the resistant phenotype to PEG stress exhibited by *GhHAI2-*, *GhAHG3-,* and *GhABI2*-silenced plants was caused by more water retention capacity, the water loss rate and RWC of detached cotton seedlings and leaves were measured. Consistently, the water loss was significantly lower in TRV2:*GhHAI2*, TRV2:*GhAHG3,* and TRV2:*GhABI2* plants compared with TRV2:00 plants (Figure 3C). In addition, silencing of *GhHAI2*, *GhAHG3,* and *GhABI2* under PEG conditions caused more RWC (93, 94, and 94%, respectively) than TRV2:00 plants (72%) and well-watered conditions (Figure 3D).

Furthermore, to determine whether the osmotic tolerance in *GhHAI2-*, *GhAHG3-,* and *GhABI2*-silenced plants is associated with the alterations in leaf physiology, we compared photosynthetic gas exchange between TRV2:*GhHAI2*, TRV2:*GhAHG3,* and TRV2:*GhABI2* plants and TRV2:00 plants. Compared with normal watering conditions and TRV2:00 plants after PEG, all three genes silenced plants showed significantly increased photosynthetic rates that were approximately double than that of control plants (Figure 3E). Similarly, significant inductions in stomatal conductance, intracellular CO_2_, and transpiration rate were also observed, indicating that *GhHAI2-*, *GhAHG3-,* and *GhABI2*-silenced plants were more tolerant to osmotic stress than control plants (Figures 3F–H). Taken together, these results indicate that silencing of *GhHAI2*, *GhAHG3,* and *GhABI2* in cotton plants significantly improved osmotic stress response.

### Silencing of *GhHAI2*, *GhAHG3,* and *GhABI2* Probably Promotes ROS Scavenging and Increases Root Biomass

**Figure 4:**
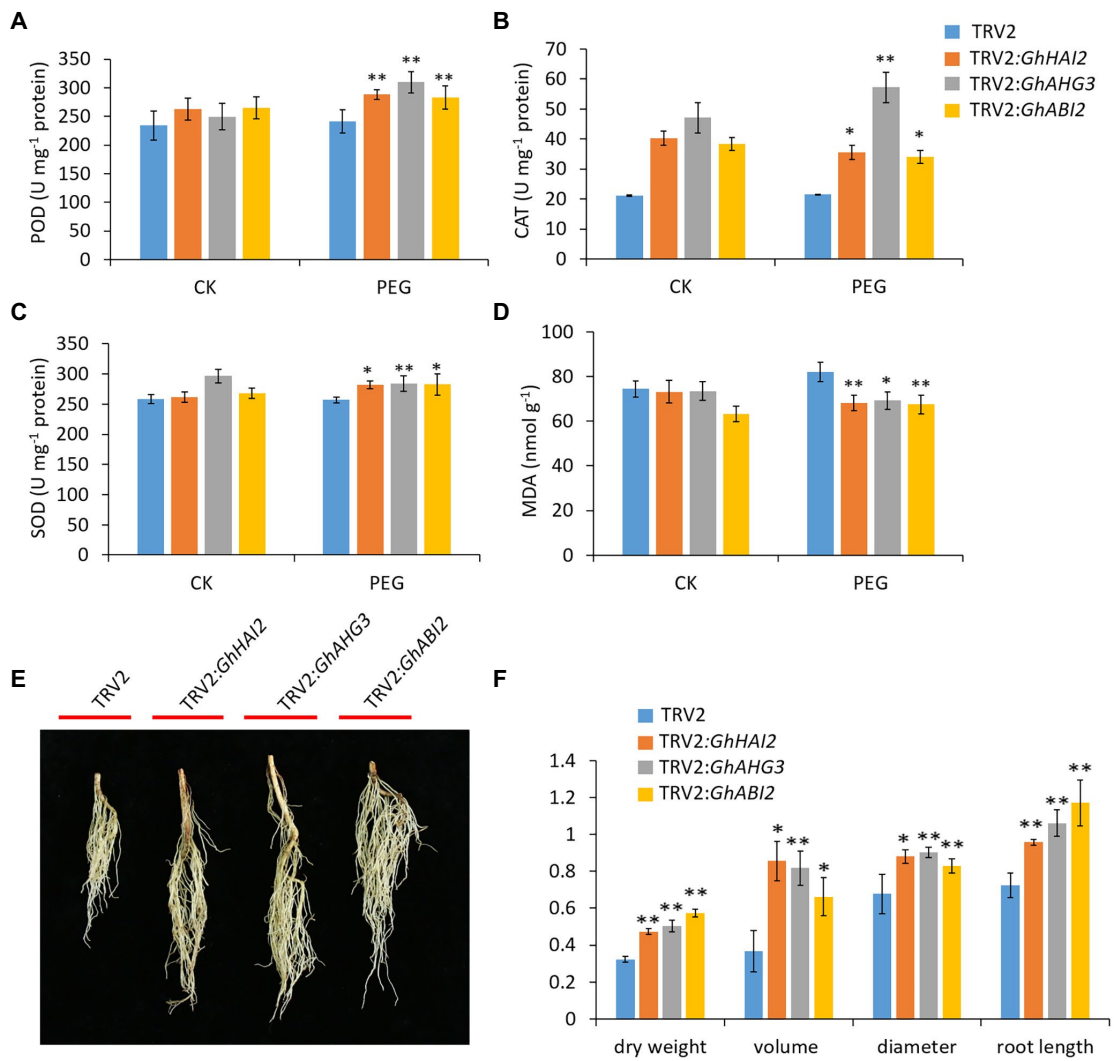
Silencing of *GhHAI2*, *GhAHG3,* and *GhABI2* promotes ROS scavenging and increases root biomass. **(A–D)** Analyses of three antioxidant enzymes; SOD, POD, and CAT activities and MDA content in TRV2:*GhHAI2*, TRV2:*GhAHG3,* and TRV2:*GhABI2* and TRV2:00 plants under normal and PEG conditions. **(E)** Root phenotype and **(F)** the root dry weight, volume, diameter, and length of TRV2:*GhHAI2*, TRV2:*GhAHG3,* and TRV2:*GhABI2* and TRV2:00 plants under PEG conditions. Bars represent means ± standard deviation of three biological replicates. Asterisks represent statistically significant difference between TRV2:*GhHAI2*, TRV2:*GhAHG3,* and TRV2:*GhABI2* and TRV2:00 plants (Student’s *t*-test; ^*^p < 0.05; ^**^p < 0.01).

In order to determine whether antioxidative mechanism is involved in *GhHAI2*, *GhAHG3,* and *GhABI2* osmotic stress response, we detected the activity of three significant antioxidant enzymes in TRV2:00 and TRV2:*GhHAI2*, TRV2:*GhAHG3,* and TRV2:*GhABI2* plants. Under both normal and PEG conditions, peroxidase (POD) superoxide dismutase (SOD) and catalase (CAT) activity increased much more in *GhHAI2-*, *GhAHG3-,* and *GhABI2*-silenced plants compared with the control plants (Figures 4A–C). Further, the MDA, which is a byproduct of lipid peroxidation under oxidative stress, was measured and compared between normal and PEG stressed plants. The MDA content was significantly lower in *GhHAI2-*, *GhAHG3-,* and *GhABI2*-silenced plants under PEG conditions compared to the control plants, indicating that silenced plants were more tolerant to osmotic stress (Figure 4D). These results showed that TRV2:00 plants were more severely damaged by ROS, while silencing of *GhHAI2*, *GhAHG3,* and *GhABI2* protected the plants from damage. Hence, *GhHAI2*, *GhAHG3,* and *GhABI2* negatively participated in the ROS scavenging pathway by increasing the activity of POD, SOD, and CAT and decreasing the MDA level in the antioxidant system under osmotic stress.

Furthermore, VIGS cotton plants exhibited significant variation in root length and biomass accumulation. We measured the root volume, dry weight, length, and diameter of *GhHAI2-*, *GhAHG3-,* and *GhABI2*-silenced and control plants under PEG conditions. The roots of *GhHAI2-*, *GhAHG3-,* and *GhABI2*-silenced plants were denser and the volume, length, and dry weight increased relative to that of control plants. Thus, under PEG conditions, the silenced plants had more vigorous root phenotypes than the control plants, which is consistent with enhanced osmotic tolerance (Figures 4E,F).

### GhHAI2, GhAHG3, and GhABI2 Interact With Cotton ABA Receptors GhPYLs

**Figure 5:**
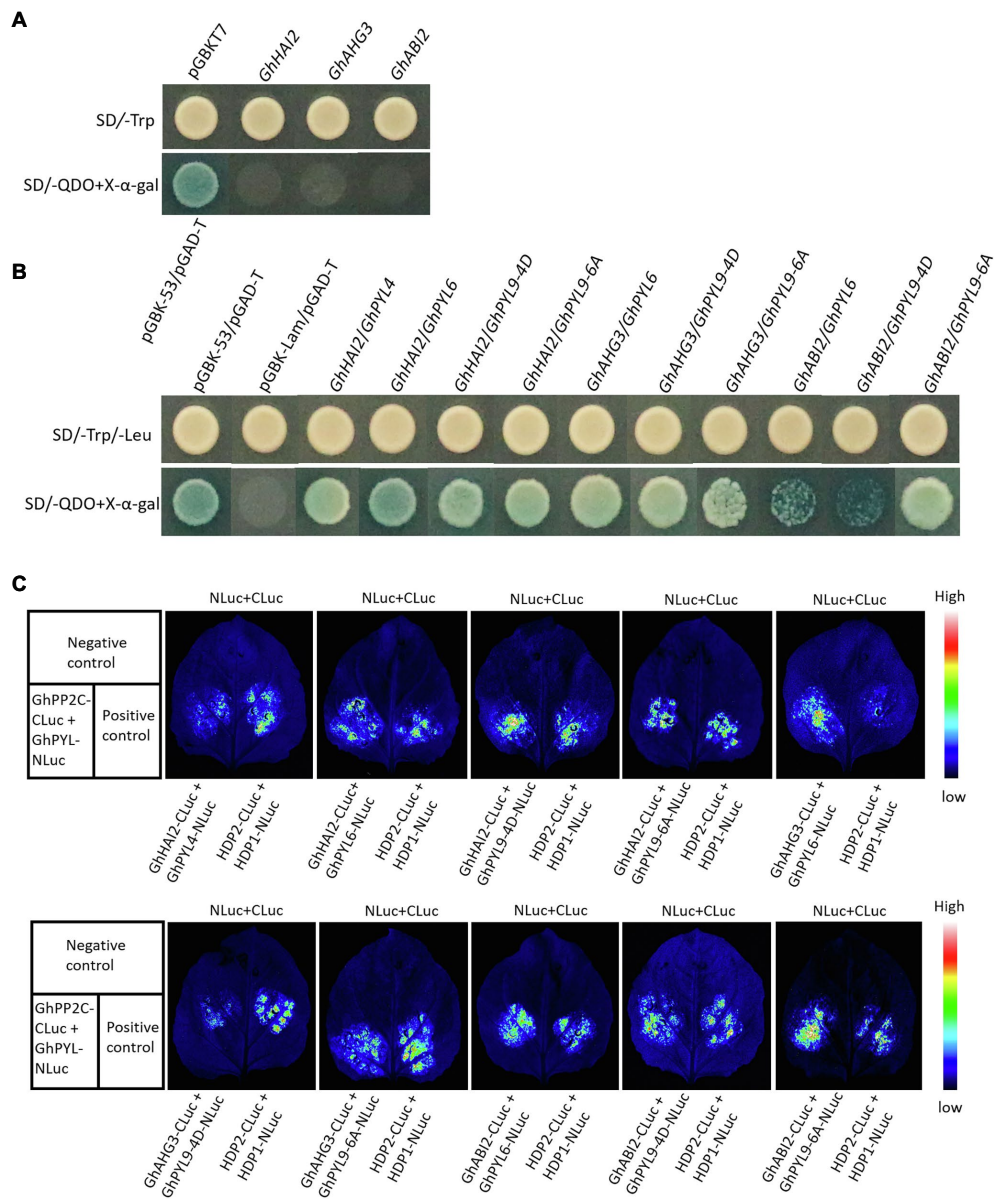
Interaction between GhHAI2, GhAHG3, and GhABI2 and ABA receptors GhPYLs. **(A)** Determination of self-activation of GhHAI2, GhAHG3, and GhABI2 in the absence of prey. **(B)** Interaction of GhHAI2, GhAHG3, and GhABI2 and GhPYLs in yeast two-hybrid assay (Y2H). The yeast strain AH109 harboring the construct pairs were plated on synthetic dropout medium either without Trp and Leu (upper panel) or without Trp, Leu, His, and Ade (SD/-QDO) containing X-α-gal (lower panel). The vectors pGBKT7-53/pGADT7-T and pGBKT7-Lam/pGADT7-T were used as positive and negative controls, respectively. **(C)** Luciferase (LUC) complementation imaging assay for analyzing the protein–protein interaction between GhHAI2, GhAHG3, and GhABI2 and GhPYLs. The specific combinations used for each interaction are indicated. The fluorescence signals represent their interaction activities.

In *Arabidopsis*, physical protein–protein interaction between clade A PP2Cs and ABA receptors PYL/PYR/RCARs is one of the principal regulatory mechanisms of ABA signaling (Santiago et al., 2012). Hence, we hypothesized that GhHAI2, GhAHG3, and GhABI2 might interact with cotton ABA receptors and therefore performed a Y2H assay. Cotton possesses 40 PYL proteins, of which we selected GhPYL4, GhPYL6, GhPYL9-4D, and GhPYL9-6A (Zhang et al., 2017) (hereafter referred to as GhPYLs). GhHAI2, GhAHG3, and GhABI2 were fused to the binding domain of GaL4 and used as bait, while GhPYLs fused to the activation domain of GaL4 and were used as prey proteins. To ensure that GhHAI2, GhAHG3, and GhABI2 alone were not able to activate the yeast reporter genes, the bait constructs were first evaluated for self-activation. The preliminary experiment revealed that there was no auto-activation of reporter genes (Figure 5A); therefore, full-length sequences of GhHAI2, GhAHG3, and GhABI2 were used to perform Y2H. The GhHAI2, GhAHG3, and GhABI2 and GhPYLs were co-transformed in a pairwise fashion into yeast. Co-transformants expressing bait and prey were able to grow on SD/−Trp/−Leu/-His/−Ade (SD/-QDO) selection medium containing X-α-galactosidase (X-α-gal), indicating the direct interaction of GhHAI2, GhAHG3, and GhABI2 and GhPYLS. Of the all co-transformants, GhAHG3/GhPYL4 and GhABI2*/*GhPYL4 did not grow on the selection medium, suggesting that GhAHG3 and GhABI2 do not directly interact with GhPYL4 (Figure 5B).

The binary protein–protein interactions were further examined *in planta* using LCI assays. Co-expression of GhHAI2-CLuc, GhAHG3-CLuc, and GhABI2-CLuc and GhPYLs-NLuc in *N. benthamiana* strongly complemented the LUC activity similar to the positive control HDP2-CLuc/HDP1-NLuc, confirming the interaction between GhHAI2, GhAHG3, and GhABI2 and GhPYLs (Figure 5C). The negative control (NLuc/CLuc) showed no visible fluorescence in tobacco leaves. Collectively, these results suggest that GhHAI2, GhAHG3, and GhABI2 interact with cotton GhPYLs and are involved in ABA signaling pathway.

### *GhHAI2, GhAHG3,* and *GhABI2* Regulate ABA-Dependent Stress-Responsive Gene Expression

**Figure 6:**
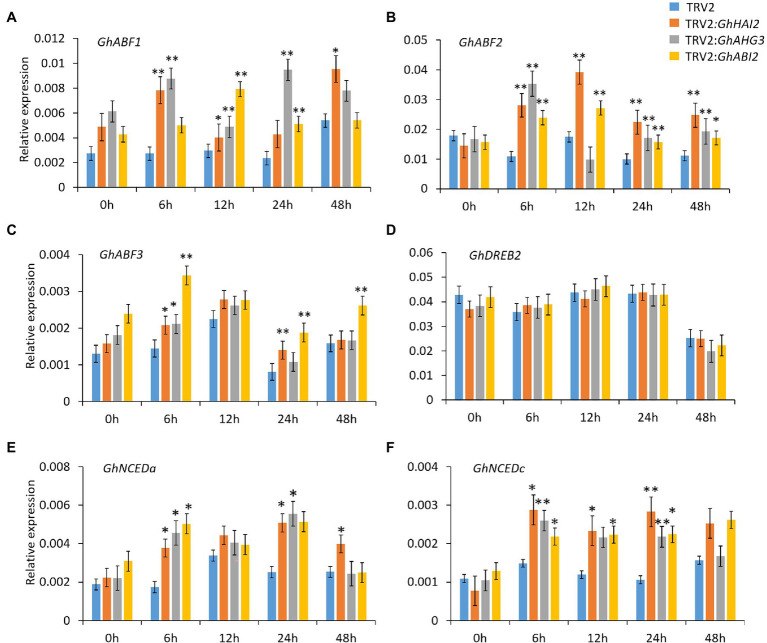
*GhHAI2*, *GhAHG3,* and *GhABI2* regulate ABA-dependent gene expression. **(A–C)** qPCR expression analysis of ABA-dependent and **(D)** ABA-independent stress-responsive genes in TRV2:*GhHAI2*, TRV2:*GhAHG3,* and TRV2:*GhABI2* and TRV2:00 plants under PEG conditions. **(E,F)** Expression levels of ABA biosynthesis genes in TRV2:*GhHAI2*, TRV2:*GhAHG3,* and TRV2:*GhABI2* and TRV2:00 plants under PEG conditions. The transcript levels were normalized by *GhHistone*. Bars represent means ± standard deviation of three biological replicates. Asterisks represent statistically significant difference between TRV2:*GhHAI2*, TRV2:*GhAHG3,* and TRV2:*GhABI2* and TRV2:00 plants (Student’s *t*-test; ^*^p < 0.05; ^**^p < 0.01).

Previous studies have revealed that the phytohormone ABA is essential for plant drought response (Xiong et al., 2006). Since clade A *PP2C* family genes have been reported to regulate plant drought stress in an ABA-dependent manner (Schweighofer et al., 2004; Antoni et al., 2012; Rodrigues et al., 2013); thus, in order to further confirm the role of *GhHAI2*, *GhAHG3,* and *GhABI2* in ABA pathway, we detected the expression level of ABA-dependent genes in *GhHAI2-*, *GhAHG3-,* and *GhABI2*-silenced cotton leaves *via* qPCR. Notably, silencing of *GhHAI2*, *GhAHG3,* and *GhABI2* exerted dramatically upregulated expression in *GhABF1, GhABF2,* and *GhABF3* (*GhABFs*) in response to osmotic stress (Figures 6A–C). However, silencing of *GhHAI2, GhAHG3,* and *GhABI2* had less effect on the induction of cotton dehydration-responsive element-binding protein 2 (*GhDREB2*) after osmotic stress (Figure 6D). ABFs and DREBs are the two major groups of transcription factors that are involved in the ABA-dependent and ABA-independent drought responses, respectively (Yoshida et al., 2014). Apparently, *GhHAI2, GhAHG3,* and *GhABI2* exerted a stronger effect on osmotic-induced expression of *GhABFs* than that of *GhDREB2*, suggesting that *GhHAI2, GhAHG3,* and *GhABI2* regulate cotton osmotic response in ABA-dependent manner. Based on this, we next examined the expression levels of ABA biosynthesis genes in *GhHAI2-*, *GhAHG3-,* and *GhABI2*-silenced cotton leaves under osmotic conditions. *GhNCED3a* and *GhNCED3c* are the orthologues of *Arabidopsis* key ABA biosynthetic gene *AtNCED3* in cotton (Jensen et al., 2013; Shang et al., 2020). Consistently, the transcripts of *GhNCED3a* and *GhNCED3c* were found to be significantly upregulated in *GhHAI2-, GhAHG3-,* and *GhABI2*-silenced plants after osmotic stress, compared to the control plants (Figures 6E,F). According to these observations, *GhHAI2*, *GhAHG3,* and *GhABI2* modulate osmotic stress by regulating ABA signaling pathway, probably by targeting ABA-dependent stress-responsive and ABA biosynthesis genes.

## Discussion

With rising water scarcity and global climate change, drought is emerging as a significant factor for limiting crop production (Zhu, 2002). Particularly, cotton production is challenged by drought stress, the reason for this may be due to more than half of the world’s cotton is produced in high water-stressed regions. Thus, there is an imperative need of understanding the molecular and genetic basis underlying cotton drought response for the development of cultivars with improved tolerance.

The plant hormone ABA plays a crucial role in many plant cellular processes, such as growth, development, and adaptation to abiotic stress (Osakabe et al., 2014). Under water deficit conditions, ABA regulates abiotic stress responses by inducing a large number of stress-related genes (Kilian et al., 2007). Previous studies have investigated the ABA signaling pathway from perception of ABA to response to stimulus (Lee and Luan, 2012). The RCAR-PP2C-SnRK2 cascade is the most well-studied ABA signaling pathway (Cutler et al., 2010). The perception of ABA by receptors and other signaling components including PP2Cs and SnRKs is critical for abiotic stress adaptation (Santiago et al., 2009; Vlad et al., 2009; Gonzalez-Guzman et al., 2012; Lim et al., 2013; Ding et al., 2015). The *Arabidopsis* group A PP2Cs have been known to function as negative regulators in ABA pathway (Saez et al., 2004; Schweighofer et al., 2004). Similarly, cotton plants presumably possess a number of drought and ABA-induced PP2Cs (Lu et al., 2019). To comprehend the importance of *PP2C* gene family in drought tolerance, we characterized three cotton clade A *PP2Cs*; *GhHAI2*, *GhAHG3,* and *GhABI2*, all of which have high sequence similarity to the *Arabidopsis* homologs *HAI2*, *AHG3,* and *ABI2*, respectively. We showed that GhHAI2, GhAHG3, and GhABI2 are nuclear-localized proteins which are responsive to PEG and ABA treatment in cotton plants. The reason for this may be due to similar functions to *Arabidopsis* group A *PP2Cs* for inducing altered phenotypes in response to ABA and drought stress, as well as their interactions with ABA signaling components.

To elucidate the role of *GhHAI2*, *GhAHG3,* and *GhABI2*, we conducted VIGS genetic analysis in cotton plants. In our phenotypic assays, *GhHAI2-*, *GhAHG3-,* and *GhABI2*-silenced plants displayed a pronounced osmotic-tolerant phenotype, which was accompanied by high RWC and increased photosynthetic gas exchange. Hence, these parameters collectively suggest that reduced expression of *GhHAI2*, *GhAHG3,* and *GhABI2* enhance the cotton plants resistance to osmotic stress.

Abiotic stresses led to oxidative damage and accumulation of MDA in plants (Alexieva et al., 2001; Hu et al., 2012). In cotton, drought stress caused the production of ROS. While, the antioxidant enzyme activity increased and regulated the ROS scavenging mechanism until the plants recovered from the stress (Ratnayaka et al., 2003). *CsATAF1-OE* plants promoted drought tolerance by enhancing the activity of antioxidant enzymes and decreasing MDA content in cucumber (Wang et al., 2018). Consistently, in our study, silencing of *GhHAI2*, *GhAHG3,* and *GhABI2* protected the plants against oxidative damage by increasing the activity of SOD, POD, and CAT and less MDA accumulation under osmotic stress.

Furthermore, we analyzed the root phenotype of VIGS plants after osmotic stress. Drought tolerance was found to be improved by root thickness, since roots can increase water absorption by encouraging root length density and growing larger root branches (Jeong et al., 2010). Under osmotic stress conditions, *GhHAI2-*, *GhAHG3-,* and *GhABI2*-silenced plants maintained their root growth, in terms of root length, volume, and root density. Additionally, the dry weight of *GhHAI2-*, *GhAHG3-,* and *GhABI2*-silenced plants was also higher than that of control plants. Hence, we propose that *GhHAI2-*, *GhAHG3-,* and *GhABI2*-mediated root modification enhance water uptake by increasing the total root surface area. Overexpression of *AtEDT1*/*HDG11* and *HYR* in rice has previously been shown to improve drought tolerance through such root-mediated system (Yu et al., 2013; Ambavaram et al., 2014). Collectively, these results suggest that silencing of *GhHAI2*, *GhAHG3,* and *GhABI2* enhances the cotton response to osmotic stress.

The interaction between PP2C and the ABA receptor PYL is the key step that triggers the downstream signaling genes to evoke ABA signaling (Fujii et al., 2009). This signaling network has been reported in different plants species such as *Arabidopsis* (Ma et al., 2009), rice (Kim et al., 2011), tomato (González-Guzmán et al., 2014), and cucumber (Wang et al., 2012). Several studies have shown the interaction between ABI1, ABI2, HABI, and AHG3 and RCAR/PYR/PYL family of ABA receptors (Ma et al., 2009; Park et al., 2009; Vlad et al., 2009). For instance, AHG3 interacts with PYL12 in response to ABA and functions specifically in seed germination and early seedling growth (Kuhn et al., 2006; Yoshida et al., 2006; Kim et al., 2011). *ABI2* has been reported to transduce ABA signals to downstream targets through selectively interplaying with PYL9/RCAR1 (Ma et al., 2009). The HAI PP2Cs interacted with PYL5 and PYL7-10 in *Arabidopsis* (Bhaskara et al., 2012). Similarly, the cotton GhPYLs are thought to interact with and inhibit GhHAI2, GhAHG3, and GhABI2, thereby activating ABA signaling pathway. Consistent with the previously reported studies, the physical interaction between GhHAI2, GhAHG3, and GhABI2 and GhPYLs was confirmed by Y2H and LCI assays. The results show that GhPYLs act as potent inhibitors of GhHAI2, GhAHG3, and GhABI2 phosphatase activity. Hence, the interaction between ABA receptors and GhHAI2, GhAHG3, and GhABI2 is necessary for the activation of downstream targets in regulating osmotic stress response.

Under water deficit conditions, plants evoke defense mechanisms *via* induction of elevated levels of ABA to moderate water consumption and enhance stress tolerance (Urano et al., 2009). The expression of drought stress-responsive genes is regulated by both ABA-dependent as well as ABA-independent pathways (Yamaguchi-Shinozaki and Shinozaki, 2006). ABFs are the bZIP family transcription factors that play pivotal role in ABA-dependent gene expression and are known as positive regulators of ABA pathway (Uno et al., 2000; Yoshida et al., 2010, 2014). Further, DREBs are large family of transcription factors that mediate drought stress through an ABA-independent pathway (Yoshida et al., 2014). Our results reveal that silencing of *GhHAI2*, *GhAHG3*, and *GhABI2* significantly increased the expression of ABA-dependent stress marker genes *GhABFs* than the ABA-independent *GhDREB2* marker gene. The expression of ABA biosynthesis genes *NCED* positively regulates the endogenous ABA levels and the transcription of both ABA- and drought-inducible genes (Iuchi et al., 2001). We found that the expression levels of the ABA biosynthesis genes *GhNCEDa* and *GhNCEDc* vary significantly between control and *GhHAI2-*, *GhAHG3-*, and *GhABI2*-silenced plants, which was consistent with the enhanced induction of *NCED3* expression in the *HAI* double and triple mutants (Bhaskara et al., 2012). These results showed that *GhHAI2*, *GhAHG3*, and *GhABI2* involved in the ABA signaling pathway by upregulating ABA-responsive genes.

## Conclusion

In conclusion, we have demonstrated that *GhHAI2*, *GhAHG3,* and *GhABI2* negatively regulate the plant adaptive response to osmotic stress *via* ABA-mediated signaling. In our VIGS genetic studies, *GhHAI2*, *GhAHG3,* and *GhABI2* displayed altered phenotypes in response to osmotic stress *via* changes in leaf physiology and root morphology and regulating ROS scavenging. Further, we have demonstrated that GhHAI2, GhAHG3, and GhABI2 act as core components of ABA signaling *via* interaction with ABA receptors. Hence, our findings provide valuable insights into the defense mechanism that occurs during osmotic stress and therefore may facilitate that these genes might be essential for drought tolerance in cotton; however, it needs further evaluation under drought stress. Furthermore, the utilization of *GhHAI2*, *GhAHG3,* and *GhABI2* mutations through genome editing techniques might also be effective.

## Data Availability Statement

The original contributions presented in the study are included in the article/Supplementary Material; further inquiries can be directed to the corresponding author.

## Author Contributions

HS conceived the study, designed the experiments, performed the experiments, interpreted the data, made figures, and wrote the manuscript. NK reviewed and edited the manuscript. LW provided assistance in qPCR analysis. XW supervised the project. All authors contributed to the article and approved the submitted version.

## Funding

This work was supported by the funding of Xinjiang Uygur Autonomous Region Major Science and Technology Project (2021A02001-3).

## Conflict of Interest

The authors declare that the research was conducted in the absence of any commercial or financial relationships that could be construed as a potential conflict of interest.

## Publisher’s Note

All claims expressed in this article are solely those of the authors and do not necessarily represent those of their affiliated organizations, or those of the publisher, the editors and the reviewers. Any product that may be evaluated in this article, or claim that may be made by its manufacturer, is not guaranteed or endorsed by the publisher.

## Supplementary Material

Supplementary Table S1 List of primer sequences used in this study.

Supplementary Figure S1 **(A)** Albino appearance on the leaves of TRV2:*GhCLA1* plants. **(B–D)** Verification of *GhHAI2*, *GhAHG3*, and *GhABI2* silencing in VIGS plants by qPCR.
