# Supplementary material for: GhHAI2, GhAHG3, and GhABI2 Negatively Regulate Osmotic Stress Tolerance via ABA-Dependent Pathway in Cotton (Gossypium hirsutum L.)
Source: Front Plant Sci. 2022 May 19;13:905181. doi: 10.3389/fpls.2022.905181 (PMC9161169; doi:10.3389/fpls.2022.905181)
Supplement: Supplementary Table S1 — List of primer sequences used in this study. [file Table_1.DOCX]

**Supplemental data**

**Table S1**. List of primer sequences used in this study

| **Primer** | **Forward sequence (5’-3’)** | **Reverse sequence (5’-3’)** |
| --- | --- | --- |
| **qPCR** | | |
| *GhHAI2-qPCR* | *TGGCGAGTGACGGTTTATG* | *ATCTCTCCTCCTCCCATCATC* |
| *GhAHG3-qPCR* | *TTAAGTTTGTGGCTGACGCT* | *CAGCGTTATCACAGTCACGA* |
| *GhABI2-qPCR* | *GGTGTCAGGTTGCGAACTAT* | *CAAACTCCTCCGATCTCAGC* |
| *GhABF1-qPCR* | *TGGGTTTGCAAGTGGAGTCA* | *CTAGCGAGCAGTGTCGTGT* |
| *GhABF2-qPCR* | *TCAGATGGGATTGGGAAGAG* | *CCTTTTCCACCGCACTGTAT* |
| *GhABF3-qPCR* | *GGAGGGTTCTTTGATGGGTT* | *AAATTGGTTGTTGCTGCTGAG* |
| *GhDREB2-qPCR* | *CTGAGTCCGCTATGGATGGT* | *GATTCCAATCCTTCAAGCCA* |
| *GhNCEDa-qPCR* | *AAGGTATCAAGGTTTGGGGTA* | *TGGAAACAAAAGCAATCAGGT* |
| *GhNCEDc-qPCR* | *GTGATGAGAGTCTCAAGAGTGTCC* | *TCAGCAAGAGCTAAATATGCGTAC* |
| *GhHistone* | *CGGTGGTGTGAAGAAGCCTCAT* | *AATTTCACGAACAAGCCTCTGGAA* |
| **Virus-Induced Gene Silencing** | | |
| *TRV2-GhHAI2* | *TGTGAGTAAGGTTACCGAATTCTCGAGAGCTGTTTTGTGTCG* | *GGGACATGCCCGGGCCTCGAGATCTCTCCTCCTCCCATCATC* |
| *TRV2-GhAHG3* | *TGTGAGTAAGGTTACCGAATTCTGTTGAGAGCTCCGGTACTA* | *GGGACATGCCCGGGCCTCGAGGTGGAGTTTGGAGTTCGCAT* |
| *TRV2-GhABI2* | *TGTGAGTAAGGTTACCGAATTCATATCAGGCACGATTGGCAG* | *GGGACATGCCCGGGCCTCGAGTCACTGGCTAGGATAAGGCA* |
| **Yeast Two-Hybrid** | | |
| *GhHAI2-pGBKT7* | *ATGGCCATGGAGGCCGAATTCATGGCGGAGATCTGTTACGG* | *CCGCTGCAGGTCGACGGATCCCCGTGGCTCTCCTTAGATCCAC* |
| *GhAHG3-pGBKT7* | *ATGGCCATGGAGGCCGAATTCATGGCTGGAGTTTGCTGTG* | *CCGCTGCAGGTCGACGGATCCCTTGATTTTCCTTCAAATCAACAACG* |
| *GhABI2-pGBKT7* | *ATGGCCATGGAGGCCGAATTCATGATGGAAGAAGTATCTG* | *CCGCTGCAGGTCGACGGATCCCTGTTTTCTTCTTAAATTTTCTC* |
| *GhPYL4-pGADT7* | *GCCATGGAGGCCAGTGAATTCATGCATGCCAATCCTCCAAA* | *CAGCTCGAGCTCGATGGATCCCTTTGCGCCTGGAGAGATTCT* |
| *GhPYL6-pGADT7* | *GCCATGGAGGCCAGTGAATTCATGCCTTCCTCTTTGCAGC* | *CAGCTCGAGCTCGATGGATCCCGGGAGATGATGACAATGATTCTTT* |
| *GhPYL9-4D-pGADT7* | *GCCATGGAGGCCAGTGAATTCATGGTGACCAATAATTATATCAC* | *CAGCTCGAGCTCGATGGATCCCCATTCTTTCGATCGGCTC* |
| *GhPYL9-6A-pGADT7* | *GCCATGGAGGCCAGTGAATTCATGAACGTGAGTAGCAGTGC* | *CAGCTCGAGCTCGATGGATCCCTCTTTCAGCTCCCAACTTTCG* |
| **Luciferase Complementation Imaging Assay** | | |
| *GhHAI2-CLuc* | *GCGTCCCGGGGCGGTACCATGGCGGAGATCTGTTACGG* | *AGTCCATTTGTTGGATCCCGTGGCTCTCCTTAGATCCA* |
| *GhAHG3-CLuc* | *GCGTCCCGGGGCGGTACCATGGCTGGAGTTTGCTGTG* | *AGTCCATTTGTTGGATCCTTGATTTTCCTTCAAATCAACA* |
| *GhABI2-CLuc* | *GCGTCCCGGGGCGGTACCATGATGGAAGAAGTATCTG* | *AGTCCATTTGTTGGATCCTGTTTTCTTCTTAAATTTTCT* |
| *GhPYL4-NLuc* | *CTCGGTACCCGGGGATCCATGCATGCCAATCCTCCAAA* | *GTACGAGATCTGGTCGACTTTGCGCCTGGAGAGATTCT* |
| *GhPYL6-NLuc* | *CTCGGTACCCGGGGATCCATGCCTTCCTCTTTGCAGC* | *GTACGAGATCTGGTCGACGGGAGATGATGACAATGATTCTTT* |
| *GhPYL9-4D-NLuc* | *CTCGGTACCCGGGGATCCATGGTGACCAATAATTATATCAC* | *GTACGAGATCTGGTCGACCATTCTTTCGATCGGCTC* |
| *GhPYL9-6A-NLuc* | *CTCGGTACCCGGGGATCCATGAACGTGAGTAGCAGTGC* | *GTACGAGATCTGGTCGACTCTTTCAGCTCCCAACTTTCG* |
| **Subcellular Localization** | | |
| *GhHAI2-GFP* | *ATTTACGAACGATAGGGTACCATGGCGGAGATCTGTTACGG* | *GCCCTTGCTCACCATGGATCCCGTGGCTCTCCTTAGATCCA* |
| *GhAHG3-GFP* | *ATTTACGAACGATAGGGTACCATGGCTGGAGTTTGCTGTG* | *GCCCTTGCTCACCATGGATCCTTGATTTTCCTTCAAATCAACA* |
| *GhABI2-GFP* | *ATTTACGAACGATAGGGTACCATGATGGAAGAAGTATCTG* | *GCCCTTGCTCACCATGGATCCTGTTTTCTTCTTAAATTTTCT* |
