# Supplementary figures and images for: GhHAI2, GhAHG3, and GhABI2 Negatively Regulate Osmotic Stress Tolerance via ABA-Dependent Pathway in Cotton (Gossypium hirsutum L.)
Source: Front Plant Sci. 2022 May 19;13:905181. doi: 10.3389/fpls.2022.905181 (PMC9161169; doi:10.3389/fpls.2022.905181)

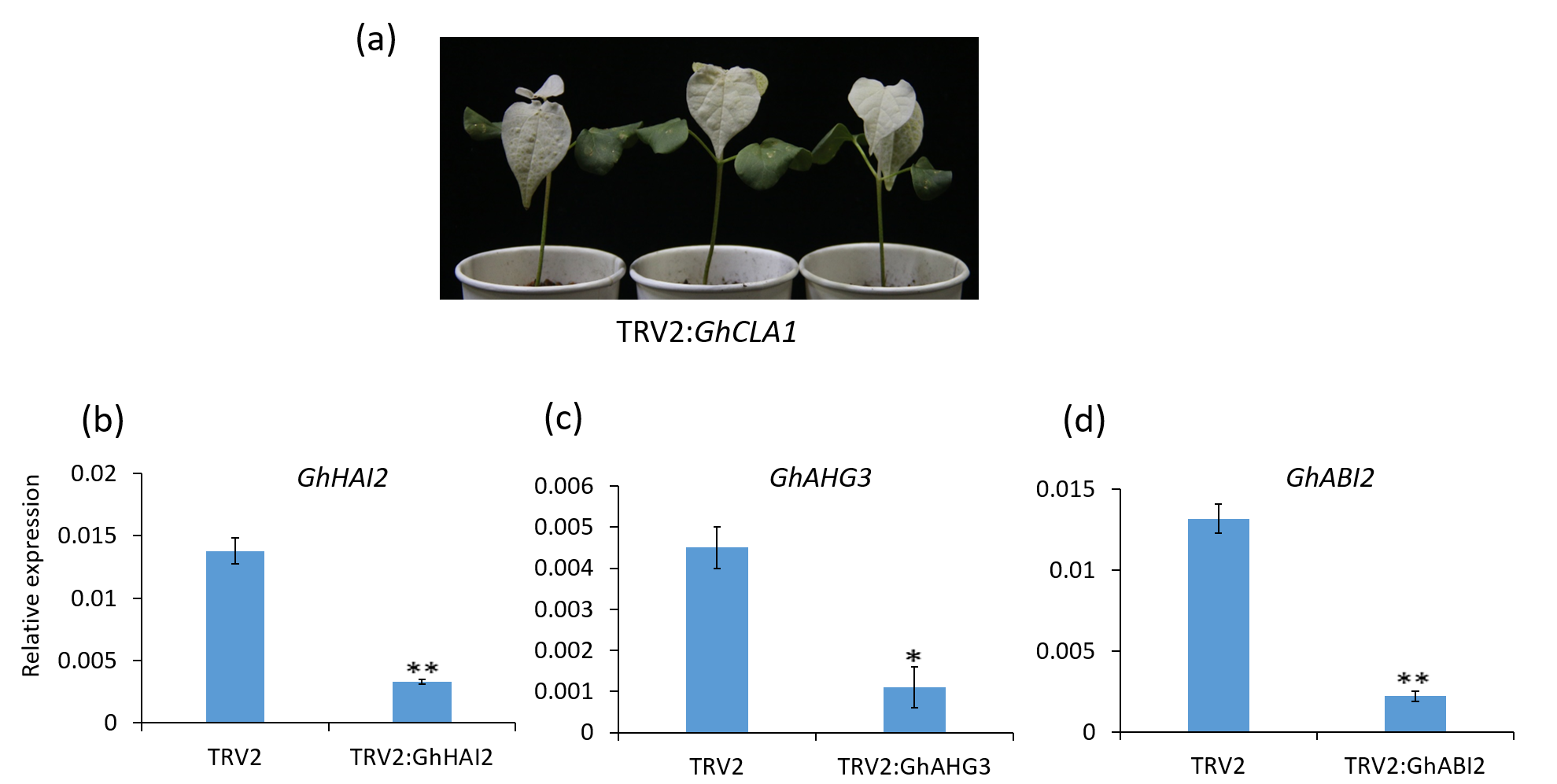

Supplement: Supplementary Figure S1 — (A) Albino appearance on the leaves of TRV2:GhCLA1 plants. (B–D) Verification of GhHAI2, GhAHG3, and GhABI2 silencing in VIGS plants by qPCR. [file Image_1.TIF]
